# Enhancing Wound Healing Through Secretome-Loaded 3D-Printed Biomaterials

**DOI:** 10.3390/gels11070476

**Published:** 2025-06-20

**Authors:** Tithteeya Rattanachot, Yogeswaran Lokanathan, Mh Busra Fauzi, Manira Maarof

**Affiliations:** 1 Department of Tissue Engineering and Regenerative Medicine, Faculty of Medicine, Universiti Kebangsaan Malaysia, Cheras, Kuala Lumpur 56000, Malaysia; p144768@siswa.ukm.edu.my (T.R.); lyoges@ppukm.ukm.edu.my (Y.L.); fauzibusra@ukm.edu.my (M.B.F.); 2 Advance Bioactive Materials-Cells UKM Research Group, Universiti Kebangsaan Malaysia, Bangi 43600, Selangor, Malaysia; 3 Ageing and Degenerative Disease UKM Research Group, Universiti Kebangsaan Malaysia, Bangi 43600, Selangor, Malaysia

**Keywords:** 3D bioprinting, biomaterials, secretome, wound healing

## Abstract

Wound healing remains a significant hurdle within the field of medical practice, especially concerning chronic and non-healing injuries. Conventional interventions, such as skin grafts, wound dressings, and biomaterials, offer structural support for the regenerated tissues but often lack the biological signaling cues essential for tissue regeneration. However, these approaches often lack the biological signals necessary to promote effective tissue repair. An emerging strategy involves incorporating cell-secreted proteins, known as the secretome, into biomaterials. The secretome contains bioactive elements such as cytokines, growth factors, and extracellular vesicles (EVs), which enhance the wound healing process. This review explores the potential of secretome-loaded biomaterials in modulating inflammation, promoting angiogenesis, and assisting in the remodeling of the extracellular matrix (ECM). Recent advancements in biomaterial engineering technology, such as 3-dimensional (3D) bioprinting, have improved the controlled delivery and bioactivity of secretome at the wound site. These gel-based biomaterials enhance wound healing by providing sustained bioactive molecule release, improving cell growth, and tissue repair. Despite these promising outcomes, limitations including variations in secretome composition and difficulties in large-scale production. Hence, secretome-loaded biomaterials offer a promising solution for wound healing, but further research is needed to optimize formulations, ensure stability, and validate clinical applications.

## 1. Introduction

Human skin is the largest organ in the body. It serves as the major point of contact between the internal and external surroundings, shielding the body from various environmental factors and preserving the homeostasis by restricting excessive electrolyte and water loss [1,2]. The skin structure composes of epidermis, dermis, and hypodermis. The functions of human skin are to protect body from external threats and from microorganism invasion to the underlying structures [3]. When the skin is injured, its natural functions and integrity can be compromised. Therefore, it is imperative to restore the structure and functions of skin as soon as possible in order to ensure the homeostasis and start the wound healing process immediately after the injury [4]. Early intervention is crucial to minimize infection risk and enhance wound healing outcomes, thereby improving patient prognosis [5].

Skin wounds or injuries can result from mechanical, chemical, or physical factors such as trauma, chronic illnesses, surgery, and burns, which provide significant challenges to the global healthcare systems, including disability and anguish. Based on the outcomes and underlying factors, skin wounds can be categorized as acute or chronic wounds [6]. Acute wounds are skin wounds that can be healed in a timely manner around 2 to 4 weeks meanwhile chronic wounds are skin wounds that take within 4 to 12 weeks to heal [7]. The normal wound healing process includes a series of phases such as haemostasis, inflammation, proliferation, and remodeling [8].

A wide range of biological and technical disciplines are included in regenerative medicine, such as gene therapy, biomaterial engineering, and stem cell biology, which converge in skin tissue engineering and wound healing to promote cellular regeneration, restore the tissue architecture, and functional recovery [9]. Among various biomaterials, hydrogels stand out for their high-water content, soft tissue mimicry, and tunable physical and chemical properties. The morphology of gels such as porosity, stiffness, and degradation profile affects the cellular interactions, nutrient diffusion, and the controlled release of therapeutic agents [10]. The ultimate goal is to create artificial organs and tissues that function well. It has the ability to address unmet clinical requirements and alleviate some of the intolerable side effects of current therapy techniques due to its immense potential to create bioengineered tissue constructs that can replace the damaged organs and tissues [11].

In recent years, the secretome, defined as the collection of bioactive molecules secreted by cells, exerts profound paracrine effects, promoting cell proliferation, angiogenesis, immunomodulation, and matrix remodeling, which are crucial for wound healing [12]. For examples, studies performed by Deng et al. and Ma et al. demonstrated that applying secretome derived by mesenchymal stem cells (MSCs) improved the healing outcomes in animal models by enhancing proliferation and migration on fibroblasts, epithelial cells, and vascular endothelial cells [13,14]. This research supports the potential of the MSC secretome over traditional stem cell therapies as a novel-free treatment for skin regeneration, such as reduced tumorigenesis risk and scalability for clinical applications [14]. In addition, a previous in vivo study also showed that secretome did not result in significant side effects, suggesting their safety for therapeutic use [15].

Concurrently, 3D bioprinting technologies enables precise and customizable fabrication of scaffolds that can mimic the tissue architecture. This approach allows for layer-by-layer deposition of bioinks composed of biomaterials, cells, and therapeutic agents to form structurally and functionally tissue constructs [16]. Based on recent study, 3D printing allows for the creation of drug-loaded, which can be designed to release therapeutic agents in a controlled manner, promoting faster healing times and improving overall treatment outcomes [17]. A previous study performed by Zhou et al. showed that 3D constructs excelled in cell viability, exceeding 95% over a five-day culture period, indicating that the bioink effectively supports the growth and proliferation of cells. Moreover, in vivo studies revealed that the constructs excelled in facilitating dermal regeneration and the use of 3D constructs has been associated with reduced infection rates [18,19]. This review aims to comprehensively explore the integration of cells-derived secretome with biomaterials and highlight the role of 3D bioprinting in enhancing the therapeutic efficacy for wound healing applications.

## 2. Skin Structure and Physiology

**Figure 1:**
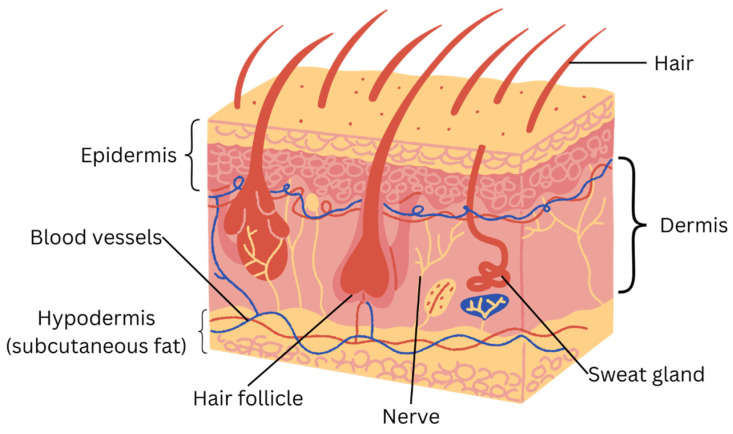
Structure of skin illustrates the outer epidermis, dermis, and hypodermis (also known as subcutaneous fat). The figure is illustrated using Canva (https://www.canva.com/).

As the largest organ in the body, skin is important because it protects it from friction and water loss, regulates body temperature, and sensory reception, which is mediated via sensory nerve endings in the skin [20]. The skin comprises the epidermis, dermis, and hypodermis (also known as the subcutaneous layer). This can be seen further in Figure 1 and Table 1, which shows the structure and key functions of the skin. The epidermis serves to shield the skin from mechanical, thermal, and chemical risks as well as microbial diseases [3]. Vitamin D for bone and calcium absorption are primarily produced by the endocrine activity of the skin, while the exocrine activity occurs when the skin secretes sweat and sebum as well as cytokines, which are the bioactive components that initiate the immune system against foreign pathogens [21]. Skin regeneration refers to the process of restoring its structure and function [22].

## 3. Wound Healing Process

Wound healing consists of four main phases, which are hemostasis, inflammation, proliferation, and remodeling. Initially, when wound occurs, the blood vessels and capillaries will constrict to reduce the blood flow. At this point, platelets are activated, and the collagen fibers then draw the platelets to form blood clots, which are made up of fibronectin, fibrin, vitronectin, and thrombospondin [4]. The inflammatory phase begins, where immune cells including neutrophils and macrophages migrate to the site of injury and mitigate the skin damage in response to exposure to chemokines. Moreover, the proliferative phase, which usually lasts a few days to weeks. It is marked by re-epithelization involves keratinocyte migration, collagen deposition, granulation tissue development, angiogenesis, and epidermal regeneration. In addition, fibroblasts and endothelial cells play critical roles by facilitating ECM production and angiogenesis, respectively [30]. Lastly, in the remodeling phase, the ECM changed into scar, during which involves the formation and rebuilding of developing tissues. The collagen is rearranged in this instance, and collagen type 1 replaces collagen type 3, which is created in the ECM, when the wound closes [4]. Table 2 summarizes the key events of each wound healing phase.

## 4. Treatments of Wound

### 4.1. Standard Treatments of Wound

According to the World Health Organization, millions of people require medical care every year due to pathological wounds [34]. Surgical treatments such as autografts, allografts, and xenografts are the three forms of skin grafts. Currently, autologous split skin graft is the standard treatment for skin abnormalities. This involves transplanting a healthy section of skin from the same individual to the wounded site. Autologous tissue enables the re-establishment of local vasculature, restores the function of epidermis, and, thus, will avoid immune rejection [34]. However, limitations include the lack of donor, risk of infection, and subsequent damage. Allografts serve as an alternative to autografts, involving the transplantation of compatible skin from another individual, and xenografts involve the use of skin samples from different animal species transplanted to humans, serving as alternatives but carrying risks such as the potential for scarring, cross-infection, and immune rejection of the transplanted tissue [34].

The topical route of administration by applying a formulated drug to the skin is widely employed over the systemic drugs. This approach avoids first pass metabolism in the liver, is easy to apply, and is well-suited for self-medication. Examples of topical formulated treatments are creams, gels, emulsions, ointments, and lotions [35]. In addition, wound infection is prevented with the usage of topical antimicrobials by either inhibiting the functions or destroying the microorganisms present in the affected area. Systemic antimicrobials have a bigger risk of bacterial resistance hence the local route is favored [34]. Table 3 shows the summary of the wound treatments, applications, and examples of the treatments.

In clinical practice, the care of cutaneous remains a major challenge, often exacerbated by factors such as infections, inflammation, and delayed healing, which interfere with normal cellular response and ECM remodeling important for tissue repair. Conventional methods, such as whole-cell treatments, have demonstrated potential but are sometimes hampered by issues with safety, inconsistent results, and exorbitant expenses. The therapeutic potential of the secretome, which is a collection of bioactive chemicals released by cells, especially mesenchymal stem cells (MSCs) has garnered increasing attention in recent years [43].

### 4.2. Alternative Treatments of Wound

Tissue engineering and regenerative medicine provide advanced strategies for wound healing by incorporating stem cells, biomaterials, and growth factors. The primary components of tissue-engineered skin include cells, biomaterials, and bioactive substances [44]. Stem cells, such as MSCs, support tissue repair by differentiating into skin-related cells such as keratinocytes and fibroblasts and releasing bioactive molecules that enhance regeneration [45]. Biomaterials, including hydrogels and scaffolds, serve as structural frameworks for facilitating cell growth. The integration of biomaterials with cells and growth factors further accelerates wound healing, particularly in chronic and non-healing wounds. This integration enhances cellular signaling, promoting angiogenesis, and facilitating ECM remodeling, contributing to the restoration of the tissue integrity. This synergy creates a bioactive microenvironment that mimics native tissue cues, leading to improved cell proliferation and faster regeneration [46]. Moreover, proteins secreted by cells, such as secretome and cytokines, play a vital role in regulating inflammation and promoting blood vessel formation, thus improving the healing process [47].

The Department of Tissue Engineering and Regenerative Medicine (DTERM) at National University of Malaysia (UKM) has developed innovative treatments for wound healing, notably MyDerm^®^, which is Malaysia’s first autologous, bi-layered tissue-engineered human skin, created using the patient’s own cells. This approach minimizes the risk of rejection and has been clinically proven to enhance wound healing, offering a permanent solution for patients with major skin loss [48]. Additionally, DTERM researchers have developed a bioactive hydrogel using 3D bioprinting technology, formulated from gelatin, polyvinyl alcohol (PVA), and genipin, which promotes rapid healing of chronic wounds. The hydrogel is biocompatible, biodegradable, and supports the regeneration of new tissue post implantation [49].

## 5. Biomaterials

**Figure 2:**
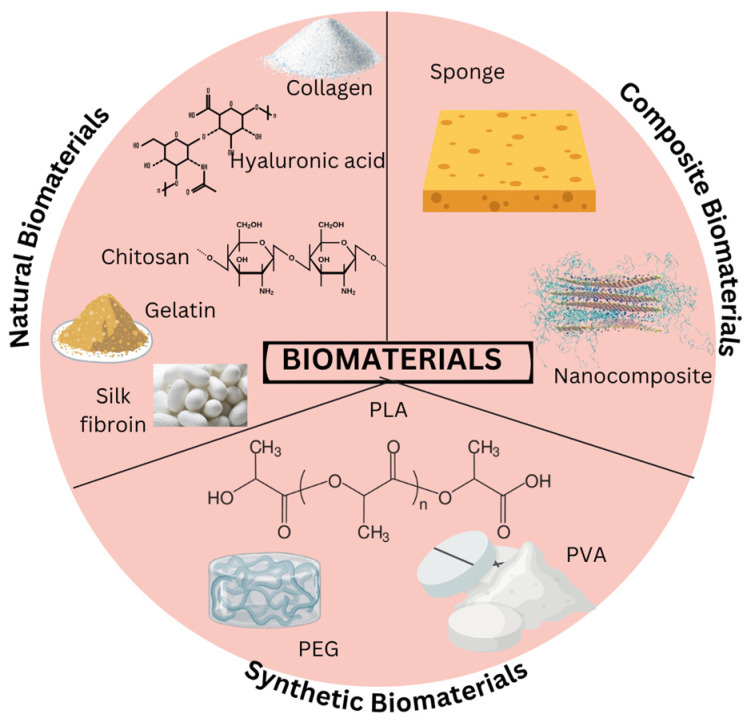
Types of Biomaterials. Biomaterials are materials designed to interact with biological systems for medical applications. They can be classified into three main types including natural, synthetic, and composite biomaterials. Natural biomaterials are derived from biological sources, synthetic biomaterials are man-made, and composite biomaterials combine natural and synthetic components. The figure is illustrated using Canva (https://www.canva.com/).

A biomaterial matrix that may facilitate the growth of physiologically active and living tissue in vitro and in vivo has been sought for more and more in recent years [50]. This can be seen further in Figure 2 and Table 4, which shows the natural and synthetic biomaterials available.

### 5.1. Naturals Biomaterials

Since natural-based biomaterials generally exhibit low immunogenicity and are biocompatible, biodegradable, and hydrophilic, they have emerged as the preferred biomaterials for tissue engineering applications. Natural biomaterials such as collagen, gelatin, and silk and polysaccharides including hyaluronic acid and chitosan have garnered significant attention in wound dressing fabrication due to the similarity to macromolecules naturally recognized by the human body. This phenomenon is referred to as ECM biomimicry [56].

### 5.2. Synthetic Biomaterials

Synthetic biomaterials are artificially synthesized macromolecules generated via chemical processes, exhibiting adjustable chemical configurations and physical characteristics and are ideal for extrusion-based bioprinting [72]. In contrast to natural ones, synthetic biomaterials exhibit lower costs, enhanced strength, and superior functional capabilities, such as tunable degradation rates and customizable mechanical properties tailored to specific tissue engineering applications. Some synthetic biomaterials can break down in the environment, and these materials can be decomposed by microorganisms or natural fluids within the living systems [62]. The biodegradable synthetic biomaterials that are frequently used consist of polylactic acid (PLA), poly(lactic-co-glycolic acid) (PLGA), and polyvinyl alcohol (PVA), which have unique advantages, such as fast degradation rates and excellent mechanical strength [62,68].

## 6. Secretome

The term ‘secretome’ describes the group of proteins, small substances, and other components that cells release into the environment. In various studies, the secretome has been used interchangeably with ‘conditioned medium’ (CM), which is defined as the culture medium containing the secretome, highlighting its therapeutic potential. This comprises several elements, including metabolites, ions, peptides, growth factors, cytokines, chemokines and ECM proteins, and EVs [73,74]. After a certain period of incubation time, the secretome is collected from the medium of cultivated cells [75].

The secretome can be harvested from a variety of human stem cell sources, but the most frequently reported ones are from mesenchymal stem cells from umbilical cord (UC-MSCs) and Wharton’s jelly (WJ-MSCs), adipose tissue-derived stem cells (ADSCs), and bone marrow-derived mesenchymal stem cells (BM-MSCs) [43]. However, challenges such as batch-to-batch variability and the need for standardized production protocols remain. MSCs can suppress pro-inflammatory T helper (Th) cell activation such as Th1 and Th17 while upregulating T regulatory (Treg) cells. This combination effect can be used to lessen transplant rejection. In addition, the secretome from MSCs has been shown to have similar immunosuppressive effects on B cells and dendritic cells [76]. UC-MSCs are mostly cultured from the umbilical vein endothelium and Wharton’s jelly, which are the two regions of the umbilical cord. The UC-MSCs secretome, also referred as conditioned medium, is the main bioactive component that facilitate in tissue regeneration [7]. Moreover, based on a previous study performed by An et al., the secretome collected from BM-MSCs can be utilized for corneal epithelial wounds [76]. A study performed by Malekzadeh et al. stated that regenerative medicine has shown potential in enhancing burn wound healing and minimizing scarring, especially in cell treatment utilizing ADSCs [77]. In addition, dermal fibroblast secretomes are easy of harvest, and expansion is simple, but their regenerative potency may be less pronounced compared to stem cell-derived secretomes.

Cells release cytokines, which are the small non-structural proteins that enable intercellular interaction via endocrine, paracrine, and autocrine mechanisms. Table 5 summarizes several growth factors that are released to stimulate the proliferation and differentiation of target cells, mainly for angiogenesis and wound remodeling processes [78].

### 6.1. Extracellular Vesicles (EVs)

Extracellular vesicles (EVs) are defined by the Minimal Information for Studies of Extracellular Vesicles (MISEV) 2018 guidelines as non-replicating particles sized approximately 30 to 150 nm for exosomes, with microvesicles and apoptotic bodies covering larger size ranges and encapsulated by a lipid bilayer. EVs, also known as tiny lipid non-replicating membrane-bound particles released by cells, often consist of bioactive molecules such as genetic materials, microribonucleic acid (microRNA), enzymes, signaling proteins, immunomodulatory factors, and growth factors [82]. EVs also comprise exosomes, microvesicle, and apoptotic bodies and are engaged in a number of biological processes, such as angiogenesis, cell migration, and intercellular cellular communication [83].

Multivesicular bodies (MVBs) contain intraluminal vesicles (ILVs) that can follow two distinct pathways. They may either fuse with lysosomes, where ILVs are degraded by hydrolase enzymes, or alternatively, MVBs can fuse with the plasma membrane, releasing ILVs into the extracellular space as exosomes [84]. Through paracrine signaling, the exosomes there perform pleiotropic actions. Research has shown that multiple lysosome-associated molecules, including tetraspanins such as CD9, CD63, and CD81, lysosomal-associated membrane protein 1, 2, and 3 (LAMP-1, -2, -3), and other markers [85]. Exosomes possess numerous beneficial characteristics that the secretome lacks, including increased stability that enables long-term preservation in vivo, the ability to modify with targeting molecules, the homing mechanism, which targets cells for internalization, and a greater capacity for protein and ribonucleic acid (RNA) loading [22].

### 6.2. Fundamental Mechanism of Secretome

**Figure 3:**
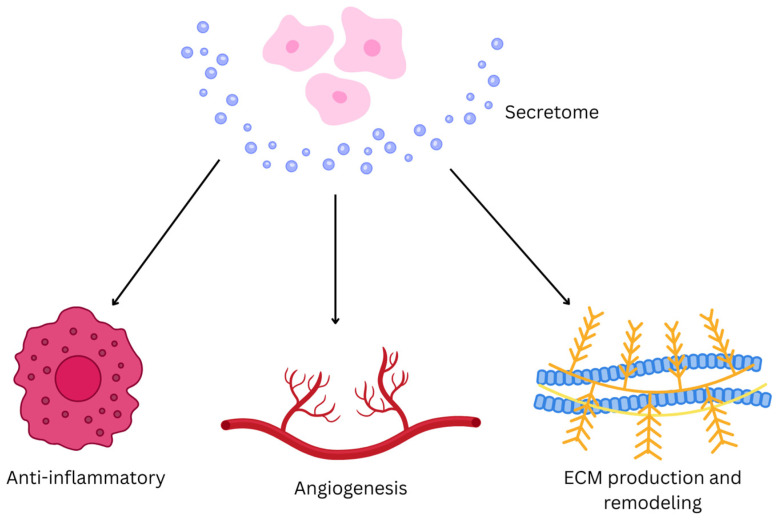
Mechanism of Action of Secretome in Wound Healing. Key mechanisms include modulation of immune response, stimulation of angiogenesis, and ECM remodeling. This facilitates wound closure, reduces inflammation, and accelerate tissue repair. The figure is illustrated using Canva (https://www.canva.com/).

Hemostasis starts as soon as wound occurs followed by chemotaxis, which is the migration of inflammatory cells by infiltration of neutrophils, macrophages, and lymphocytes. Secretome-derived bioactive molecules, including exosomes, cytokines, and growth factors, activate key signaling pathways such as the phosphoinositide 3-kinase (PI3K) PI3K/Akt and janus kinase/signal transducer and activator of transcription (Jak-STAT), promoting cellular responses crucial for healing [83]. Growth factors and cytokines present in secretome can promote tissue remodeling, cell migration, and proliferation. They also contribute in reducing the inflammatory phase while encouraging angiogenesis and re-epithelization. Growth factors like vascular endothelial growth factor (VEGF) and TGF-β promote the formation of new blood vessels, ensuring adequate oxygen and nutrient supply to the tissue, while activation of PI3K/Akt signaling pathway can enhance fibroblasts, keratinocytes, and vascular cell migration and proliferation [78,86]. In addition, lymphocytes start the production of TNF, granulocyte colony-stimulating factor (G-CSF), and granulocyte macrophage-colony stimulating factor (GM-CSF). In contrast, bFGFs are essential for granulation tissue formation, stimulating fibroblast proliferation, migration, and ECM production. Epidermal growth factor (EGF) accelerates re-epitheliazation by promoting keratinocyte proliferation and migration. It also enhances fibroblast activity, contributing to tissue remodeling and wound contraction. Platelet-derived growth factor (PDGF) will promote the recruitment and proliferation of fibroblasts and smooth muscle cells [75]. Figure 3 illustrates that by delivering secretome to the injury site, the wound healing process is accelerated through enhanced cell recruitment, proliferation, and ECM remodeling, making it a promising therapeutic strategy for tissue regeneration.

### 6.3. Uses of Secretome

There are several key usages of secretome including wound healing, cell-free therapy, regenerative medicine, biomaterial integration, and inflammation modulation. Furthermore, secretome reduces the hazards of stem cell transplantation, which carries the risk of infection including tumor growth and immunological rejection, by providing a cell-free substitute for conventional stem cell therapies [22]. In addition, growth factors including EGF, hepatocyte growth factor (HGF), and bFGF are among the constituents of secretome, and they are essential for tissue regeneration and repair. In addition, combinations of secretome and biomaterials are said to be increase the therapeutic effectiveness in clinical situations. Research has demonstrated that secretome can regulate inflammatory reactions by fostering a healing environment that has anti-inflammatory properties [65].

When treating degenerative disorders, the MSC secretome has been shown to have therapeutic qualities and can be utilized as a substitute for cells without compromising the effectiveness of the therapeutic impact, primarily through paracrine signaling mechanism [87]. Cell-to-cell communication can be directly mediated via the secretome, or it can lead nearby cells to release bioactive substances. Like vaccines and monoclonal antibodies, secretome treatments are allogeneic and can be established ahead of time as a ready-made treatment for a variety of illnesses [87,88]. Additionally, the secretome may be easily stored and transported by freeze-drying or lyophilising it, unlike cell-based products that need cryopreservation [89].

Several in vitro and in vivo studies have been performed to evaluate the benefits of secretome in wound healing applications. In vitro evaluations usually focus on analyzing the capability of cells to migrate and proliferate. The migration assay method and the scratch assay are commonly employed to evaluate the migratory capacity of cells. This involves comparing the rates of wound closure between control and treatment groups. Additionally, the expression levels of MMP2 and MMP9, which are the proteins that play key roles in cell migration processes, can be analyzed to further validate the effects on cell migration [90]. The WST-1 test, MTT assay, and proliferation assay methods are commonly employed to evaluate the capacity of cells to proliferate. The WST-1 and MTT assays measure the metabolic activity as an indirect indicator of viable cells, while proliferation assays specifically track the DNA synthesis or cell number over time. These assays aim to determine whether secretome administration enhances cell viability. Additionally, the angiogenesis assay serves as a complementary method to investigate the potential of secretome to promote angiogenesis [91]. Research conducted by Miranda et al. reported that the secretome derived from stem cells can effectively enhance the migration of keratinocytes and dermal fibroblasts. Similarly, Sera et al. observed an increase in the expression of Ki67, which is a marker associated with cell proliferation, following treatment with secretome. Furthermore, the application of the secretome was shown to modulate tube formation in in vitro angiogenesis assays [92].

Considering a variety of components in the secretome that can promote the regeneration of tissues and organs, the secretome holds great promise for tissue engineering and regenerative medicine field. While the cell-free therapy is comparatively safe for human health, the secretome comprises cell-secreted substances that have been shown to provide therapeutic effects for regenerative treatment applications without cellular components. Several recent studies going through modifications in the secretome using biomaterials have shown a notable improvement in the regeneration result and a significant acceleration closure of wound [75].

## 7. Secretome-Loaded Biomaterials

The assortment of biological substances related to biomaterials comprises distinct active agents including proteins such as growth factors, cytokines, chemokines, and EVs, which are sourced from cells such as MSCs within a biomaterial framework. When the secretome exits the biomaterials and enter the adjacent tissues, it reveals its ability to connect with immune cells such as macrophages and lymphocytes. Such forms of interaction might encourage a decrease in an inflammatory response, advancement of healing, and the fostering of the engagement of the implant biomaterials within the host tissues [93]. Through the encapsulation of the secretome within biomaterials such as alginate microcapsules, researchers can facilitate a regulated liberation of these bioactive compounds [94]. This approach can sustain therapeutic levels over an extended duration and diminish the probability of swift degradation or loss of bioactivity, which often occurs through enzymatic breakdown, hydrolysis, or oxidation in the wound microenvironment [95]. Both in vitro and in vivo findings show that the secretome diminishes the foreign body response tied to implanted biomaterials. This method demonstrates potential for several uses, spanning tissue engineering, regenerative medicine, and enhancing implant compatibility by reducing negative immune reactions [96]. Table 6 shows the detailed several studies utilizing the secretome into biomaterials.

## 8. 3D Bioprinting Technology

While conventional scaffold fabrication techniques have contributed to the development of biomaterials for secretome delivery, their limitations in spatial control, uniformity, and biomimicry highlight the need for advanced approaches leading to the emergence of 3D bioprinting as a promising solution. Three-dimensional bioprinting has revolutionized skin tissue restoration by enhancing wound healing and regeneration while addressing limitations of conventional methods include solvent casting, freeze-drying, and electrospinning, which are often time-consuming and complex [101]. This technology enables replacement of damaged tissues with bioengineered constructs by incorporating cellular components and biomolecular, including growth factors, into the provisional bioscaffolds. These constructs facilitate tissue maturation and remodeling, improving regenerative outcomes. By leveraging advanced imaging such as X-ray, computed tomography (CT) scans, and magnetic resonance imaging (MRI), 3D bioprinting ensure precise identification of the anatomy and physiology of defective tissues [102].

The operational principle of 3D bioprinting encompasses the sequential depositing of bioink in layers, guided by the computer-aided design (CAD), allowing for the rapid fabrication of scaffolds with controlled porosity. Additionally, it ensures that the bioscaffolds exhibit excellent mechanical and structural properties such as tensile strength and elasticity of the scaffolds. Among the various 3D bioprinting techniques, extrusion-based bioprinting, magnetic bioprinting, stereolithography, and photolithography are the most recognized 3D bioprinting techniques [101].

Moreover, the integration of 3D bioprinting with advanced materials has led to the development of multifunctional wound dressings capable of responding to the wound environment. For instance, smart 3D-bioprinted hydrogel dressings have been designed to provide controlled release of therapeutic agents, antimicrobial properties, and real-time monitoring of wound conditions. These innovations not only enhance the healing process but also offer personalized treatment options tailored to patient needs [103].

An earlier research endeavor established a bioink conducive to 3D-printing by incorporating nano hydroxyapatite and deproteinised bovine bone into a collagen matrix. A porous scaffold was subsequently produced utilizing a 3D printer, aiming to achieve a bone replacement material that closely resembles the structural and compositional characteristics of natural bone. Previous investigation also revealed that poly(lactic-co-glycolic acid) (PLGA)/collagen nanofibrous membranes significantly enhanced tendon-osseointegration within the lateral cortex, with histological scores and biomechanical strength markedly improved in the treatment groups compared to controls, such as ultimate failure load for both groups were 38.6 ± 4.7 nm and 27.4 ± 3.9 nm, *p* < 0.05, suggesting that the composite polymer possesses a pronounced efficacy in facilitating tendon remodeling in an experimental rabbit model [62]. With its abundance of natural cell-binding sites, enzymatic degradation potential, and temperature-dependent gelation, collagen is a potential natural biomaterial [27].

A previous study by Guo et al. utilized a fabrication of a collagen-based scaffold incorporating hydroxyapatite (HAP) and BM-MSCs using 3D bioprinting showed a significant advancement in bone tissue engineering aimed at improving healing outcomes [104]. The combination demonstrated suitable rheological properties for 3D extrusion printing, resulting in a composite scaffold that enhanced mechanical strength compared to pure collagen scaffolds. The compressive moduli exceeded those of pure collage scaffolds, which can cover nearly 100% of their original structures, indicating a robust structure capable of withstanding physiological loads [104].

Furthermore, a study by Niu et al. reported the fabrication of sodium alginate/gelatin/collagen (SA/Gel/C) scaffolds with micro-nano porosity using 3D bioprinting. In vivo studies resulted in wound coverage improvement of approximately 85% within 14 days, and the cell viability was around 90% at day 6, indicating the ability of the scaffolds to support cell survival and proliferation in a conducive environment [105].

### 8.1. Integration of Secretome with 3D-Printed Biomaterials

The integration of secretome with 3D-printed biomaterials represents an advance strategy for enhancing tissue regeneration due to the synergistic effects of biological activity and structural support [93]. The components of secretome including EVs, cytokines, and growth factors can be embedded in 3D-printed biomaterials to create bioactive constructs with targeted and sustained release profiles [106]. For instance, secretome-loaded hydrogels or scaffolds have been fabricated using extrusion-based bioprinting, allowing for enhanced cell proliferation, angiogenesis, and ECM remodeling [107]. This combination enables the construction of tissue environments in supporting cellular functions and improving the regenerative outcomes of skin wounds, bone defects, and neural injuries [45]. Moreover, 3D bioprinting offers control of secretome distribution, enabling precise deposition of bioactive cues where regeneration is needed the most [108].

In a study by Bari et al., the extrusion-based 3D printing was used to fabricate poly (ε-caprolactone) (PCL) scaffolds co-printed with alginate hydrogel containing lyophilized BM-MSCs secretome. The constructs demonstrated prolonged release of EVs and proteins, resulting in significantly enhanced osteoinductive potential and mineralized matrix deposition in vitro, proving their efficacy for bone tissue engineering [107].

In addition, Liu et al. utilized collagen/silk fibroin scaffolds embedded with secretome from bFGF-pretreated UC-MSCs. In a canine traumatic brain injury model, the bioactive scaffold promoted neural regeneration and improved neurobehavioral recovery, attributed to enhanced matrix remodeling and anti-inflammatory signaling [109].

### 8.2. Preclinical and Clinal Applications of Secretome-Loaded 3D-Printed Biomaterials

A study utilized methacrylated hyaluronic acid (HAMA) bioink to 3D print wound dressings embedded with MSC-derived small EVs. These dressings facilitated controlled release of EVs leading to improved wound epithelialization, angiogenesis, and innervation in a diabetic ulcer mouse model [110]. The in vitro analyses demonstrated that both dermal fibroblasts and endothelial cells could internalize the small EVs, which significantly stimulated cell proliferation and migration. The in vivo experiments resulted in improved wound closure rates where the patches facilitated a controlled released of small EVs over a period of 7 days, which contributed to enhanced healing. In addition, it showed significant improvements in re-epithelialization, indicating that the small EVs effectively promoted the regeneration of the epithelial layer and enhanced blood vessel formation, known as neovascularization in the wound area [110].

Furthermore, 3D-bioprinted scaffolds composed of collagen, silk fibroin, and secretome from bFGF-pretreated MSCs from human umbilical cord were fabricated in a canine traumatic brain injury model. These scaffolds demonstrated enhanced biodegradation, mechanical properties, and excellent biocompatibility. In vitro assessments demonstrated significantly improved cell proliferation and differentiation, as evidenced by higher MTT assay values in the treated group, indicating a more effective biomimetic neuronal network formation. In vivo assessments showed notable improvements in motor function and reduced neuronal loss, as well as enhanced angiogenesis and reduced inflammation. The outcomes showed in accelerated brain tissue regeneration and improved functional recovery [109].

**Figure 4:**
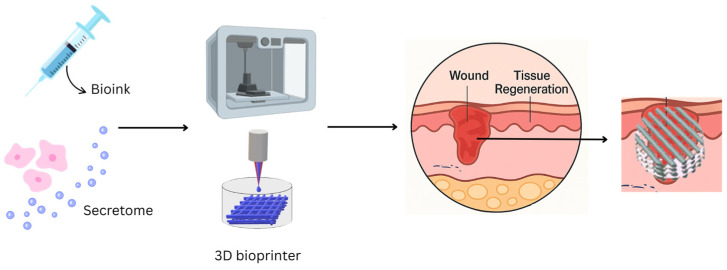
Schematic representation of 3D bioprinting in wound healing applications. The illustration outlines the workflow of 3D bioprinting technology, including bioink formulation with therapeutic agents such as secretome, and layer-by-layer deposition onto the wound site. The construct can be applied directly to wounds. The figure is illustrated using Canva (https://www.canva.com/).

While the majority of research is still in the preclinical stage, emerging clinical applications are beginning to surface. One clinical study investigated the use of a secretome-loaded hydrogel for chronic wound healing, reporting faster closure rates and improved granulation tissue formation compared to control group. It supports the translational potential of secretome-based therapies [111]. Figure 4 illustrates the core components and sequence of 3D bioprinting for fabricating biomaterials used in regenerative wound healing, which offers advantages in structural precision, controlled release, and enhanced healing outcomes [112].

A phase 1 clinical trial (NCT06217627) was conducted in Beirut, Lebanon, in June 2023, evaluating the effects of the UC-MSC secretome on skin rejuvenation. The participants received the secretome injections in various sites. Primary outcomes included assessments of wrinkle severity using standardized scales and concentrations of growth factors found in the secretome. Secondary outcomes involved the histological examinations of skin biopsies before treatment and after 12 months. Collagen, keratin, elastin, blood vessels membrane, and fibroblast nuclei were evaluated [113].

Another phase 1/2 clinical trial (NCT05921058) was conducted in March 2023 by Universitas Sebelas Maret in Indonesia assessed the safety and efficacy of MSC secretome therapy in patients with systemic lupus erythematosus (SLE). The treatment group received hypoxia-preconditioned MSC secretome infusions on days 1 through 7, and subsequently on days 14, 21, and 28. Primary outcomes included changes in inflammatory markers such as erythrocyte sedimentation rate (ESR), IL-6 as well as the Mexican Systemic Lupus Erythematosus Disease Activity Index (MEX SLEDAI) scores [114].

A patent titled “Kit for the Reconstitution of a Cell-Free Biomedical Device for Use in Regenerative Medicine, Biomedical Device thus Reconstituted and Related Synthesis Process” (WO 2025/061827 A1) composed of hyaluronic acid and heparin, which is rehydrated using MSC-secretome rich in growth factors and chemokines. The biomaterials enable controlled release of secretome-derived bioactive factors at the injury site, with the release rate modulated by adjusting the ratio of heparin to hyaluronic acid, as heparin has a higher affinity for secretome proteins [115].

### 8.3. Ideal 3D-Bioprinting Materials Requirements

An ideal 3D bioprinting material must exhibit good printability, biocompatibility, good mechanical properties, biodegradability, and sterilization stability. Printability refers to the ability of bioinks to be systematically deposited with high precision, ensuring the dimensional accuracy of the final construct [116]. Inkjet bioprinting depends on the viscosity of the bioinks, whereas micro-extrusion bioprinting can utilize more viscous materials to preserve the original 3D form post-printing, with cross-linking used to stabilize the final form. In addition, biocompatibility is another critical factor, ensuring that printed biomaterials do not elicit any detrimental local or systemic responses [96]. The biocompatibility of a material is predominantly determined by its chemical composition, structural morphology, surface characteristics, surface charge, and mechanical properties of the materials. Enhancements such as altering the surface, including the modification of material topology as well as the regulation of surface hydrophilicity and hydrophobicity. In addition, biomimetic approaches are employed to develop materials that closely replicate native tissue structures and functions.

**Figure 5:**
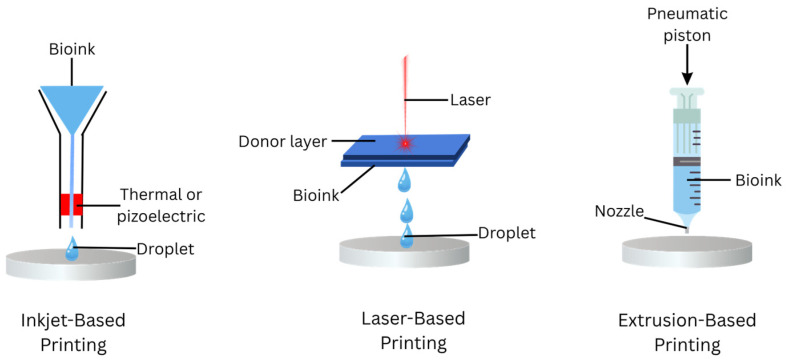
Schematic illustration of 3D bioprinting techiques used for biomaterials fabrication. The diagram compares inkjet-based, laser-based, and extrusion-based techniques. Inkjet-based printing dispenses droplets of low-viscosity bioink through thermal or pizoelectric activation. Laser-based printing employs laser pulses to transfer bioink from a donor layer to a substrate. Extrusion-based printing utilized pneumatic or mechanical force to push bioink hrough a nozzle. The figure is illustrated using Canva (https://www.canva.com/).

Mechanical strength is essential for printed constructs to endure external forces while preserving the original shape and structure. Depending on the application, materials must be engineered to exhibit appropriate mechanical properties, sometimes using sacrificial materials for temporary support [96,112]. Once implanted, the biomaterials should degrade concurrently with cellular growth, development of ECM, and tissue development, ensuring non-toxic byproducts that are easily metabolized and excreted. Ideally, degradation rates should align with the healing timeline ranging from weeks for skin and soft tissue to several months for bone and tendon regeneration [116]. Furthermore, sterilization is a crucial step in biomedical applications, with various techniques such as autoclaving, electron beam or gamma ray irradiation, ethylene oxide exposure, as well as immersion in ethanol, to effectively eliminate all varieties of microorganisms. Materials used in 3D bioprinting must be compatible with at least one sterilization technique while maintaining their structural and functional integrity [112]. The differences among these techniques are visually summarized in Figure 5.

### 8.4. 3D-Bioprinting Techniques

Bioprinting represents a swiftly evolving discipline distinguished by a multitude of biologically oriented deposition and assembly mechanisms. These systems incorporated an array of methodologies including direct writing, microstamping, photolithography, laser writing, electro-printing, microfluidics, stereolithography, extrusion, and inkjet deposition. The optimal bioprinter is required to fulfill particular system specifications, encompassing elevated resolution, substantial throughput, the proficiency to simultaneously dispense diverse materials, biocompatibility, preservation of cellular viability, consistency in process execution, and the capacity to regulate the dispensability of bioinks. Organ printing methodologies, predicated on core operational principles, can be executed through the utilization of various technological approaches, such as extrusion, inkjet, and systems based on laser deposition. A detailed summary of the bioprinting techniques, methods, benefits, and limitations is given in Table 7.

## 9. Challenges and Future Perspectives

Current regulatory and manufacturing challenges, including batch-to-batch consistency and scalability, are being addressed to facilitate clinical trials of secretome-loaded bioprinted scaffolds. These variables can lead to inconsistent therapeutic outcomes, which the standardization and reproducibility are difficult across different research and clinical settings. In addition, the optimal method for loading and releasing the secretome in 3D-printed scaffolds remains a challenge. The controlled kinetics of bioactive molecules release while maintaining the bioactivity is crucial yet technically complex. Moreover, regulatory hurdles also complicate the clinical translation, as these products lie between medical devices and biologics. In Malaysia, oversight may involve both the Medical Device Authority (MDA) and the National Pharmaceutical Regulatory Agency (NPRA), respectively. Additionally, GMP compliance for secretome production and ISO certification for 3D-printed devices are required for quality control and sterility.

The latest breakthroughs in the field of biomaterials and regenerative medicine present transformative approaches that could greatly benefit the healing of wounds. The utilization of artificial intelligence in the design of biomaterials represents a swiftly advancing domain in which machine learning algorithms, such as random forest models or deep neural networks enhance material characteristics to achieve superior biocompatibility, degradation kinetics, and mechanical strength. An additional compelling domain of investigation pertains to innovative combinations of secretome, wherein constituents of the secretome are synergistically combined with various bioactive components, including peptides, small molecules, or engineered EVs. These integrative approaches seek to augment the regenerative properties of the secretome through the concurrent targeting of various wound healing pathways. Progress in the development of patient-tailored biomaterials and customized secretome compositions depends on genetic and biochemical wound assessments might yield enhanced therapeutic outcomes, especially for individuals suffering from chronic wounds.

## 10. Conclusions

Biomaterials enriched with the secretome denote a considerable enhancement in the area of wound healing, delivering a thorough approach for tissue regeneration. By harnessing the regenerative capabilities of secretome, which is abundant in cytokines, growth factors, and EVs, biomaterials function as proficient delivery systems. These composite structures facilitate the healing process by regulating inflammatory responses, stimulating angiogenic processes, and encouraging the deposition of ECM while concurrently offering structural reinforcement customized to the specific requirements of the wounds. Nonetheless, a multitude of challenges persists, such as the need for standardization in secretome collection, the assurance of consistency across different batches, and the navigation of regulatory obstacles, including compliance with Good Manufacturing Practice (GMP), securing FDA approval for clinical use, and implementing rigorous quality control and safety testing protocols. Addressing these obstacles through collaborative efforts among researchers, healthcare professionals, and governing bodies will be crucial for effectively implementing these innovations in medical environments. In short, biomaterials that incorporate secretome can enhance wound healing.

## Figures and Tables

**Table 1:** Key functions of the skin and their structural basis. This table outlines the major physiological roles of the skin such as protection, sensation, thermoregulation, excretion, immune function, and fluid balance along with the corresponding skin structures responsible for each function.

Function	Structure	Description	References
Protection	Epidermis	Serves as a physical barrier, protecting against environmental threats such as pathogens, chemicals, and physical injuries	[3,23]
Sensation	Dermis	Enables the perception of touch, pressure, temperature, and pain, facilitating interaction with the environment	[24,25]
Thermoregulation	Dermis and hypodermis	Contributes to body temperature regulation through mechanisms such as sweating and modulation of blood flow	[24,25,26,27,28]
Excretion	Dermis	Aids in the excretion of metabolic waste products through sweat glands	[26]
Immune function	Epidermis and dermis	Serves as an immunological barrier detecting and responding to pathogens	[3,29]
Fluid balance	Epidermis	Prevents excessive water loss, thus maintaining proper hydration and fluid balance within the body	[24]

**Table 2:** Phases of wound healing and key events. This table summarizes the stages of wound healing comprises hemostasis, inflammation, proliferation, and remodeling along with cellular events that occur in each phase.

Phases	Key Events	References
Hemostasis phase	Platelets are activated, and the collagen fibers then draw the platelets to form blood clots which are made up of fibronectin, fibrin, vitronectin, and thrombospondin	[4,8]
Inflammation phase	i. Immunological barrier against microbes is formed ii. Secretion of growth factors including platelets derived growth factor (PDGF), transforming growth alpha (TGF-α), transforming growth factor beta (TGF-β), fibroblast growth factor (FGF), and insulin-like growth factor-1 (IGF-1)	[4,31]
Proliferative phase	i. Collagen deposition—activated fibroblast first shifts to the wound site and generates ECM ii. Granulation tissue development iii. Angiogenesis mediated by endothelial cells. iv. Epidermal regeneration	[4,32]
Remodeling phase	i. Scar tissue formation ii. Collagenase breaks down extra collagen fibers, collagen reorganizes, and enlarged capillaries recede as part of the maturation process	[33]

**Table 3:** Types of wound treatments and their applications. This table categorizes common wound treatment approaches and describes their specific clinical applications.

Treatments	Types	Description	Examples	References
Full-thickness Skin Graft (FTSG)	Autograft	Involves transplanting both the epidermis and entire dermis layers of the skin	Abdomen	[36]
Split-thickness Skin Graft (STSG)	Autograft	Skin taken from the patient’s own body and can be used for large wounds, burns, and ulcers	Thigh skin grafts	[37]
	Allografts	Skin obtained from a human donor	Cryopreserved cadaveric skin	[38]
	Xenografts	Skin derived from an animal source used as a temporary biological dressing	Porcine skin grafts	[38]
Wound Dressings	Hydrocolloid	Forms a gel upon contact with wound exudate, maintaining moisture. Best for wounds with minimal exudate	Duoderm	[39]
	Hydrogel	Provides moisture to dry wounds. Best for wounds with minimal exudate	Intrasite Gel, Aquaform	[40]
	Transparent Film	Thin, adhesive, and waterproof dressing that allows wound visualization while preventing contamination	Tegaderm	[41]
	Antimicrobial Dressing	Contains agents like iodine to reduce bacterial load and prevent infection	Iodoflex	[42]
	Foam Dressing	Absorbs moderate to heavy exudate, maintains a moist environment, and provides cushioning	Mepilex	[37]

**Table 4:** Summary of natural and synthetic biomaterials for tissue engineering and wound healing. This table compares the biomaterials in terms of source, properties, limitations, and typical uses in wound healing and tissue regeneration.

Biomaterials	Types	Source	Key Properties	Limitations	Applications	References
Natural biomaterials	Collagen	Human and animal ECM (bovine, pig, mouse, marine)	Biocompatible and can be used in 3D-printed scaffolds for bone or tendon repair	Lack mechanical strength and requires modifications	Widely used in tissue engineering.	[51,52,53]
	Gelatin	Derived from partial hydrolysis of collagen	Biodegradable, biocompatible, and low immunogenicit	Poor viscosity and mechanical strength at high temperatures	Skin repair, tissue engineering, GelMA for cell encapsulation	[53,54,55]
	Silk	Extracted from silkworm cocoons	Biocompatible, promotes wound healing phases, antibacterial with nanodiamond	Less effective against Gram-positive bacteria	Wound dressings and tissue engineering	[50,56,57]
	Hyaluronic acid	Found in ECM of connective and epithelial tissues	Enhances cell adhesion, proliferation, differentiation, and water soluble	Immunoevasive in pathogens	Wound healing, 3D bioprinting, and viscosity enhancer	[54,58,59,60]
	Alginate	Extracted from brown algae	Biocompatible, biodegradable, and supports cell growth	Poor cell adhesion	Wound healing and tissue regeneration	[61]
	Chitosan	Crustacean shells	Antibacterial, modifiable, bioadhesive, and enhances drug delivery	Limited mechanical strength	Hydrogels, nanofibers, and drug delivery	[56,62,63]
Synthetic biomaterials	PLA	Plant-based from lactic acid monomers	Biodegradable thermoplastic and supports bone regeneration	Weak mechanical properties	Bone scaffolds and 3D printing with additives	[62,64,65]
	PVA	Synthetic polymer	Biocompatible, non-toxic and water soluble	Poor haemostasis, antibacterial activity, and hydrophilicity	Hemostatic dressings and wound healing with modifications	[66,67]
	Polyglycolic acid (PGA)	Synthetic polymer	Fast degradation and high mechanical strength	Produced acidic degradation products	Tissue engineering	[68]
	PEG	Synthetic polymer	Tunable, cell-encapsulating, and non-toxic	Requires modification to optimize its performance	Scaffolds and diabetic wound healing	[54,56,69]
Composite biomaterials	Polysaccharide-bioceramic composites	Natural polysaccharides with ceramic phases	Enhanced bioactivity, osteoconductivity, and mechanical reinforcement	Brittleness and complex fabrication	Bone tissue engineering and scaffold reinforcement	[70]
	Nanostructured polymer composites	Polymers with nanoparticles or nanofillers	Improved mechanical, thermal, and biological properties	Cost and scale-up challenges	Advanced wound healing, scaffold fabrication, and drug delivery	[71]

**Table 5:** Key growth factors released and their roles in wound healing. This table lists essential growth factors involved and highlights their specific roles in angiogenesis, cell proliferation, and matrix remodeling.

Growth Factors	Role in Wound Repair	References
IL-1, IL-6, IL-8	Promotes angiogenesis of wounds and regeneration of epithelium	[79,80]
PDGF	Increased fibroblast and endothelial cell proliferation, migration, and invasion ability	[79]
TGF	Promoted ECM remodeling, ultimately promotes wound healing and reduces scar formation	[80]
bFGF	Migration and proliferation of fibroblasts	[81]
VEGF	Proliferation and migration of endothelial cells, acceleration of wound angiogenesis, promotes migration of fibroblasts	[79]
EGF	Promotes proliferation of fibroblasts	[79]

**Table 6:** Secretome-loaded biomaterials for regenerative therapy. This table provides an overview of biomaterials integrated with the cell-derived secretome, focusing on their compositions, delivery strategies, and therapeutic outcomes in regenerative medicine.

Study	Biomaterials	Model	Findings	Limitations	References
Hyaluronic Acid Sponge with MSC Secretome	Hyaluronic acid sponge	In vivo (psoriasis skin model)	Porous sponge enables sustained release of MSC secretome, promoted 50% increase in keratinocyte proliferation, angiogenesis, and inflammation needed for dermal wound repair	Clinical efficacy not yet validated, limited to psoriasis model	[97]
GelMA-PEGDA Hydrogels with MSC Secretome	GelMA and poly(ethylene glycol) diacrylate (PEGDA) hybrid hydrogels	In vitro (hyperglycemic human dermal fibroblasts)	Restored proliferation and migration of hyperglycemic fibroblasts to more than 85% wound closure, potential for diabetic wound healing	In vivo efficacy and long-term effects not assessed	[98]
Alginate/ECM Hydrogel Patch with hMSC Secretome	Alginate combined with decellularized ECM	In vivo (rat skin wound model)	Accelerated wound closure rate of 92% by day 14, improved angiogenesis, and increased in collagen deposition	Limited to skin wound model	[94]
Photopolymerizable GelMA Hydrogels with hADSC Secretome	GelMA hydrogels	In vitro (scratch assay, tube formation)	Enhanced fibroblast migration by 65% and angiogenesis, tunable release of secretome components	Requires in vivo validation, potential variability in hydrogel formulations	[99]
Fibrin Glue with MSC secretome	Fibrin-based hydrogels	In vivo (rat intestinal anastomosis model)	Improved anastomotic healing, increased granulation tissue and collagen deposition, and promoted 1.8 fold angiogenesis	Focused on intestinal model, broader applications need exploration	[100]

**Table 7:** 3D-bioprinting techniques. This table summarizes various 3D-bioprinting methods including extrusion, inkjet, and laser-based bioprinting, highlighting their working principles, advantages, and disadvantages for tissue engineering.

Bioprinting Techniques	Description	Advantages	Disadvantages	Examples of Biomaterials	References
Extrusion-Based Printing	Utilizes a fluid-dispensing mechanism and robotic system to extrude bioink as continuous cylindrical filaments	i. Compatible with a wide range of biomaterials with varying viscosities. ii. Structural support for high-viscosity materials	Increased mechanical stress reduces cell viability	GelMA-alginate and gelatin-fibrin	[108,117,118]
Inkjet-Based Printing	Deposits bioink onto a substrate either as a continuous flow or discrete droplets using electronically controlled ink cartridges	i. High resolution and reproducibility ii. Cost-effective and high cell viability (>90%) iii. Enable precise placement of multiple cell types	Limited to low viscosity bioinks that can be ejected through a nozzle	Collagen, fibrinogen-alginate, hyaluronic acid	[53,119,120]
Laser-Based Bioprinting	Uses laser-induced forward transfer to deposit bioink without physical contact, minimizing cellular stress	i. High cell viability (>95%) ii. Compatible with various bioinks and viscosities iii. No mechanical shear stress on cells	Expensive and complex control systems limit accessibility	Collagen-gelatin, alginat-MSC secretome	[53,118]

## Data Availability

No new data were created or analyzed in this study.

## Abbreviations

The following abbreviations are used in this manuscript:

EVs	Extracellular vesicles
ECM	Extracellular matrix
3D	Three-dimensional
PDGF	Platelets derived growth factor
TGF-α	Transforming growth alpha
TGF-β	Transforming growth beta
FGF	Fibroblast growth factor
IGF-1	Insulin-like growth factor-1
ROS	Reactive oxygen species
MMPs	Matrix metalloproteinases
FTSG	Full-thickness skin graft
STSG	Split-thickness skin graft
MSCs	Mesenchymal stem cells
PVA	Polyvinyl alcohol
GAG	Glycosaminoglycan
PLA	Polylactic acid
PLGA	Poly(lactic-co-glycolic acid)
PEG	Poly(ethylene glycol)
CM	Conditioned medium
Th	T helper cells
IL	Interleukin
VEGF	Vascular endothelial growth factor
EGF	Epidermal growth factor
MVEs	Multivesicular endosomes
ILVs	Intraluminal vesicles
MVBs	Multivesicular bodies
RNA	Ribonucleic acid
CT	Computed tomography
MRI	Magnetic resonance imaging
CAD	Computer-aided design
